# When Chocolate Seeking Becomes Compulsion: Gene-Environment Interplay

**DOI:** 10.1371/journal.pone.0120191

**Published:** 2015-03-17

**Authors:** Enrico Patrono, Matteo Di Segni, Loris Patella, Diego Andolina, Alessandro Valzania, Emanuele Claudio Latagliata, Armando Felsani, Assunta Pompili, Antonella Gasbarri, Stefano Puglisi-Allegra, Rossella Ventura

**Affiliations:** 1 Santa Lucia Foundation, Rome, Italy; 2 Department of Psychology and Center “Daniel Bovet,” Sapienza University, Rome, Italy; 3 Department of Applied Clinical Science and Biotechnology, University of L'Aquila, Coppito, Italy; 4 CNR, Institute of Cellular Biology and Neurobiology, Rome, Italy; University of Lübeck, GERMANY

## Abstract

**Background:**

Eating disorders appear to be caused by a complex interaction between environmental and genetic factors, and compulsive eating in response to adverse circumstances characterizes many eating disorders.

**Materials and Methods:**

We compared compulsion-like eating in the form of conditioned suppression of palatable food-seeking in adverse situations in stressed C57BL/6J and DBA/2J mice, two well-characterized inbred strains, to determine the influence of gene-environment interplay on this behavioral phenotype. Moreover, we tested the hypothesis that low accumbal D2 receptor (R) availability is a genetic risk factor of food compulsion-like behavior and that environmental conditions that induce compulsive eating alter D2R expression in the striatum. To this end, we measured D1R and D2R expression in the striatum and D1R, D2R and α1R levels in the medial prefrontal cortex, respectively, by western blot.

**Results:**

Exposure to environmental conditions induces compulsion-like eating behavior, depending on genetic background. This behavioral pattern is linked to decreased availability of accumbal D2R. Moreover, exposure to certain environmental conditions upregulates D2R and downregulates α1R in the striatum and medial prefrontal cortex, respectively, of compulsive animals. These findings confirm the function of gene-environment interplay in the manifestation of compulsive eating and support the hypothesis that low accumbal D2R availability is a “constitutive” genetic risk factor for compulsion-like eating behavior. Finally, D2R upregulation and α1R downregulation in the striatum and medial prefrontal cortex, respectively, are potential neuroadaptive responses that parallel the shift from motivated to compulsive eating.

## Introduction

Eating disorders are caused by environmental and genetic factors and their complex interactions [1, 2]. However, there are few gene- environment studies on human eating disorders [2] and animal studies that have examined environmental and genetic factors in compulsive food seeking and intake [3–6].

Stressful experiences interact with genetic factors and increase the risk for addictive behaviors inducing changes in the corticostriatal dopamine (DA) and norepinephrine (NE) signals that mediate motivational salience attribution [7–9]. Mounting evidence has implicated dopamine receptors in motivated behavior [10–14] and D2Rs in the proclivity toward compulsion-driven behaviors, such as addiction [15–17].

Inbred strains of mice provide valuable models for studying the interaction between genetic and environmental factors [18]. C57Bl6 ⁄ J (C57) and DBA2⁄ J (DBA) mice are among the most frequently studied inbred strains with regard to psychobiology because they are characterized by clear differences in a number of behavioral responses. The functional and anatomical characteristics of their brain neurotransmitter systems, as well as the behavioral outputs to reinforcing and aversive stimuli, have been examined extensively in these strains, thus providing important information on how the response of different neural systems to the same environmental stimuli is related to genetic background, leading to different (or also opposite) behavioral outputs [19–23]. In particular, C57 and DBA mice are commonly used in drug abuse research because of their different sensitivity to the incentive properties of, and differential responses to, addictive drugs, such as alcohol, psychomotor stimulants, and opiates [7, 20, 21, 24–31]. Moreover, with regard to psychopathological endophenotypes [32–34], disparities between C57 and DBA mice in D2R-associated phenotypes appear to depend on gene-environment interactions [35–37].

DBA mice are poorly responsive to rewarding stimuli compared with C57 mice, a state that is highlighted by chronic stressful experiences, increasing drug responsiveness in DBA/2 mice [24]. Thus, we hypothesize that chronic stress exposure (caloric restriction) induces a similar motivational drive toward palatable food in the DBA strain. We examined compulsive eating with regard to conditioned suppression of palatable food-seeking under adverse conditions [38], in C57 and DBA mice. Food restriction in rodents is commonly considered a stressful conditions leading to, among other effects, altered sensitization of brain reward systems and affecting the attribution motivational salience processes [8, 24, 39–42]. Moreover, it has been reported that greater sensitization of the reward system can lead to excessive intake of highly palatable food [38, 43, 44], and repeated stimulation of reward pathways through highly palatable food may lead to neurobiological adaptations that make the intake behavior more compulsive [45]. Of the environmental factors that influence some eating disorders, the availability of seductive foods is the most obvious [45] and it has been demonstrated that different foods establish different levels of compulsive behaviors [45, 46]. Of all palatable foods, chocolate has been showed to have rewarding properties in animals [9, 47–49], and it is the food most typically associated with reports of food craving in humans. Thus, chocolate craving and addiction have been proposed in humans [50].

Because caloric restriction is a stressful experience [24], animals were placed on a moderate food-restriction schedule [38], and because pre-exposure to palatable food is a significant factor in eating disorders [51], they were also pre-exposed to chocolate. Overeating shares several neural substrates with compulsive drug-seeking [52, 53]. Based on the function of DA receptors in drug- and food-related behaviors [17, 51, 54, 55], we measured D1R and D2R subtype levels in the caudate putamen (CP), nucleus accumbens (NAc), and medial prefrontal cortex (mpFC) and alpha-1 adrenergic receptors (α1Rs) in the mpFC because prefrontal NE is required for compulsive food-seeking [38] and α1Rs mediate motivation and drug-reinforcing effects [56–58].

We found that exposure to environmental conditions induces compulsion-like eating behavior, depending on the genetic background. This behavioral pattern was linked to decreased availability of accumbal D2Rs. Moreover, such exposure upregulated D2Rs and downregulated α1Rs in the striatum and medial prefrontal cortex, respectively, of compulsive animals.

These findings confirm the function of gene-environment interplay in the expression of compulsive eating and support the hypothesis that low accumbal D2R availability is a “constitutive” genetic risk factor of compulsion-like behavior. Thus we propose that D2R upregulation and α1R downregulation in the striatum and medial prefrontal cortex, respectively, are potential neuroadaptive responses that parallel the shift from motivated to compulsive eating.

## Materials and Methods

### Animals

Male C57BL/6JIco and DBA/2J mice (Charles River, Como, Italy), 8–9 weeks old at the time of the experiments, were group-housed and maintained on a12-h/12-h light/dark cycle (light on between 7 AM and 7 PM), as described [9, 38]. All experiments were conducted per the Italian Law (Decreto Legislativo no. 116, 1992) and the European Communities Council Directive of November 24, 1986 (86/609/EEC) regulating the use of animals for research. All experiments of this study were approved by the ethics committee of the Italian Ministry of Health and therefore conducted under license/approval ID #: 10/2011-B, according with Italian regulations on the use of animals for research (legislation DL 116/92) and NIH guidelines on animal care. Adequate measures were taken to minimize the pain and discomfort of animals. Control groups were subjected only to the “brief pre-exposure” to chocolate (2 days); Stressed groups were subjected to the “pre-exposure” to chocolate, “caloric restriction” and “brief pre-exposure” to chocolate before the conditioned suppression procedure started (see above for methodological details).

All experiments were conducted during the light phase.

### Conditioned Suppression Procedure

The apparatus for the conditioned suppression test has been previously described [38]. A Plexiglas cup (3.8 cm in diameter) was placed in each chamber and fixed to prevent movement: 1 cup contained 1 g of milk chocolate (Kraft) (Chocolate-Chamber, C-C), and the other cup was empty (Empty Safe- Chamber, ES-C).

Briefly, the procedure was as follows: from Day 1 to Day 4 (training phase), mice (Control, Stressed groups for each strain) were placed individually in the alley, and the sliding doors were opened to allow them to enter both chambers freely and explore the entire apparatus for 30 minutes. On Day 5, the animals were exposed to light-foot shock pairings. Acquisition of the conditioned stimulus (CS) (light)-shock association was established in a different apparatus, comprising a 15×15×20 cm Plexiglas chamber with a black-and-white-striped pattern on 2 walls (to differentiate it from the conditioned suppression apparatus) and a stainless steel grid floor through which the shocks were delivered. The light was produced by a halogen lamp (10W, Lexman) under the grid floor that was turned on for 5, 20-sec periods every 100 sec.; in each period, after the light had been on for 19 sec, a 1-sec 0.15-mA scrambled foot shock was delivered. This session of light-shock association lasted for 10 min and was followed by a 10-min rest period, after which another identical 10-min light-shock association session was administered; overall, the mice received 10 light-foot shock pairings in a 30-min session. On Days 6–8, the mice were left undisturbed in their home cage. On Day 9, conditioned suppression of chocolate-seeking was measured in a test session (conditioned suppression test day), in which the mice had access to chocolate in 1 of the 2 chambers in which chocolate had been placed during the training phase. In the chamber that contained chocolate (C-C), the CS (light) was presented according to the paradigm for the light-foot shock association (except for the 10-min rest period, which was eliminated). The light was produced by a halogen lamp under the grid floor that was turned on for 20-sec periods every 100 sec. This session lasted 20 min; overall, the mice received 10 20-sec periods in a 20-min session.

The test session began with the first 20-sec burst of light. The time that was spent in each of the 2 chambers was recorded throughout the session. All experiments were performed in experimental sound-attenuated rooms that were indirectly lit by a standard lamp (60 W). For all behavioral tests, data were collected and analyzed using “EthoVision” (Noldus, The Netherlands), a fully automated video-tracking system. The acquired digital signal was then processed by the software to extract “time spent” (in seconds) in the chambers, which was used as raw data for preference/aversion scores in each sector of the apparatus for each subject.

Two groups of mice for each strain were used in the conditioned suppression experiment: control (Control n = 6) and stressed (Stressed n = 8).

### Experimental Procedure

**Fig 1:**
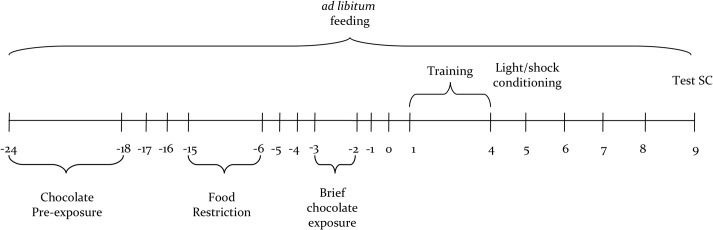
Timeline of Experimental Procedure. (See Methods for details.)

The experimental procedure is depicted in Fig. 1.

### Pre-exposure to chocolate

Animals in the stressed groups (Stressed C57 and Stressed DBA) were exposed to chocolate for 7 days until 18 (from day -24 to day -18, Fig. 1) days before the conditioned suppression procedure began. Mice were “randomly” isolated daily for 4 hours; milk chocolate and standard food were delivered *ad libitum*. Two days after the end of this schedule (day -15, Fig. 1), mice in the Stressed group were subjected to caloric restriction (food restriction, FR).

### Caloric Restriction

Mice were assigned to a feeding regimen: they either received food *ad libitum* (Control groups) or were subjected to a food restricted regimen (FR, Stressed groups). In the caloric restriction condition, food was delivered once daily (07.00 p.m.) in a quantity adjusted to induce a loss of 15% of the original body weight. In the *ad libitum* condition, food was given once daily (07.00 p.m.) in a quantity adjusted to exceed daily consumption [38].

Animals were placed on a moderate FR schedule [29] for 10 days (from day -15 to day -6, Fig. 1), until 6 days before the conditioned suppression procedure began (day 1, Fig. 1). Six days before the training phase started, the animals were returned to *ad libitum* feeding in order to rule out any effects of dietary deficiency on the conditioned suppression test day.

### Brief pre-exposure to chocolate

To prevent any unspecific novelty responses to chocolate in the groups that were not subjected to the “pre-exposure” condition described above (Control groups), both control and Stressed groups, were exposed to chocolate on the same schedule for 2 days, 2 days before the conditioned suppression procedure started (“brief pre-exposure”).

### Chocolate intake and animal weight

Chocolate intake during the various phases of the conditioned suppression procedure (pre-exposure, training, test) was measured, and the animals weight was recorded. Mice were weighed on: the first day of the experiment (before the experimental procedure began), the training phase days, and the day of the conditioned suppression test.

### Dopaminergic and noradrenergic receptors expression in Control and Stressed DBA mice

α1R, D1R and D2R receptors expression in 3 brain regions [mpFC (α1R, D1R, D2R); NAc (D1R, D2R); and CP (D1R, D2R)] was measured by western blot in control (Control DBA n = 6) and stressed animals (Stressed DBA n = 8), the same groups used in the conditioned suppression experiment.

### Dopaminergic and noradrenergic receptor expression in naïve C57 and DBA mice

Baseline D1R, and D2R receptors expression in the mpFC, NAc, and CP as well as baseline α1R in the mpFC was measured in naïve animals of both strains [naïve C57 (n = 6) and naïve DBA (n = 6)] by western blot. This experiment was performed in animals subjected neither to environmental conditions (pre-exposure to chocolate, FR) nor to the conditioned suppression procedure (naïve groups) in order to test the hypothesis that low striatal D2 receptors availability is a genetic risk factor of food compulsion-like behavior.

### Western blotting

The mice were sacrificed by decapitation, and the brains were removed 1 h after the conditioned suppression test, except for the naïve groups. The prefrontal, accumbal, and striatal tissue was dissected and kept in liquid nitrogen. Punches of the mpFC, NAc, and CP were obtained from frozen brain slices as reported [59] (S1 Fig.) and stored in liquid nitrogen until the day of the assay. Each tissue sample was homogenized at 4°C in lysis buffer (20 mM Tris (pH 7.4), 1 mM EDTA, 1 mM EGTA, 1% Triton X-100) with protease inhibitor cocktail (Sigma-Aldrich, St. Louis, MO, USA).

The tissue extract was centrifuged at 12,000 g at 4°C for 30 min. The supernatant was treated in the same way as the tissue extract. Finally, The supernatant was removed and stored at 80°C.

Protein content was measured by Bradford assay (BioRad Laboratories, Hercules, CA, USA).

The mpFC, NAc, and CP were analyzed using, 60 ug, 30 ug, and 30 ug, respectively, of each protein sample after addition of sample buffer (0.5 M Tris, 30% glycerol, 10% SDS, 0.6 M dithiothreitol, 0.012% bromophenol blue) and boiling for 5 min at 95°C. Proteins were separated by electrophoresis on 10% acrylamide/bisacrylamide gels and transferred electrophoretically to nitrocellulose membranes, which were then blocked for 1 h at 22°C–25°C in Tris-buffered saline (in mM: 137 NaCl and 20 Tris-HCl, pH 7.5), containing 0.1% Tween 20 (TBS-T) and 5% low-fat milk.

The membranes were incubated with primary antibodies [rabbit anti-dopamine D1 (Immunological Sciences) and rabbit anti-dopamine D2 receptor (Immunological Sciences), diluted 1:800 in TBS-T with 5% low-fat, or rabbit anti-alpha1-adrenergic receptor (Abcam), diluted 1:400 with 1% low-fat milk overnight at 4°C. After being washed extensively in TBS-T, the membranes were incubated for 1 h at room temperature (22°C–25°C) with HRP-linked secondary antibodies [anti-rabbit IgG diluted 1:8000 (immunological sciences) in TBS-T with 5% low-fat milk] and developed with ECL-R (Amersham). The signals were digitally scanned and quantified using densitometric image software (imagej 64), normalized to tubulin.

### Statistics

#### Conditioned Suppression experiment

For the conditioned suppression test, statistical analyses were performed for the time (sec) spent in the center (CT), in the chamber that contained chocolate (C-C) and in the empty safe chamber (ES-C) during the training phase (overall mean of 4 days of training) and on the day of the conditioned suppression test. The data were analyzed using repeated-measures ANOVA, with 2 between-group factors (strain, 2 levels: C57, DBA; treatment, 2 levels: Control, Stressed) and 1 within-group factor (chamber, 3 levels: CT, C-C, ES-C). Mean time spent in the C-C and ES-C chambers was compared using repeated-measures ANOVA within each group. Between-group comparisons were analyzed when appropriate by one-way ANOVA.

#### Chocolate intake and weight

Chocolate intake during training (overall mean of 4 days) and on the conditioned suppression test day was analyzed by two-way ANOVA (strain, 2 levels: C57, DBA; treatment, 2 levels: Control, Stressed). Chocolate intake during the pre-exposure phase was analyzed by one-way ANOVA (strain: Stressed C57, Stressed DBA). The animals weight was also recorded on the first day of the experiment (before the experimental procedure), during the training phase, and on the day of the conditioned suppression test. The data were analyzed by two-way ANOVA (strain, 2 levels: C57, DBA; treatment, 2 levels: Control, Stressed).

#### Dopaminergic and noradrenergic receptors expression in Control and Stressed DBA mice

D1R and D2R expression in the mpFC, NAc, and CP and D1R, D2R, and α1R levels in Stressed DBA versus Control DBA were analyzed by one-way ANOVA (treatment, 2 levels: Control DBA, Stressed DBA).

#### Dopaminergic and noradrenergic receptors expression in naïve C57 and DBA mice

D1R and D2R expression in the mpFC, NAc, and CP and D1R, D2R, and α1R levels in naïve C57 and DBA animals (naïve C57, naïve DBA) were analyzed by one-way ANOVA (strain, 2 levels: C57, DBA).

## Results

### Conditioned suppression experiment: Food-seeking behavior in Stressed DBA mice

In order to assess the interplay between genetic background and environmental conditions exposure on the expression of compulsive eating behavior, the time spent in C-C and ES-C on the different phases (training and test) of the conditioned suppression procedure shown by Stressed and Control groups of both strains was assessed (Control C57, Control DBA, Stressed C57, Stressed DBA).

**Fig 2:**
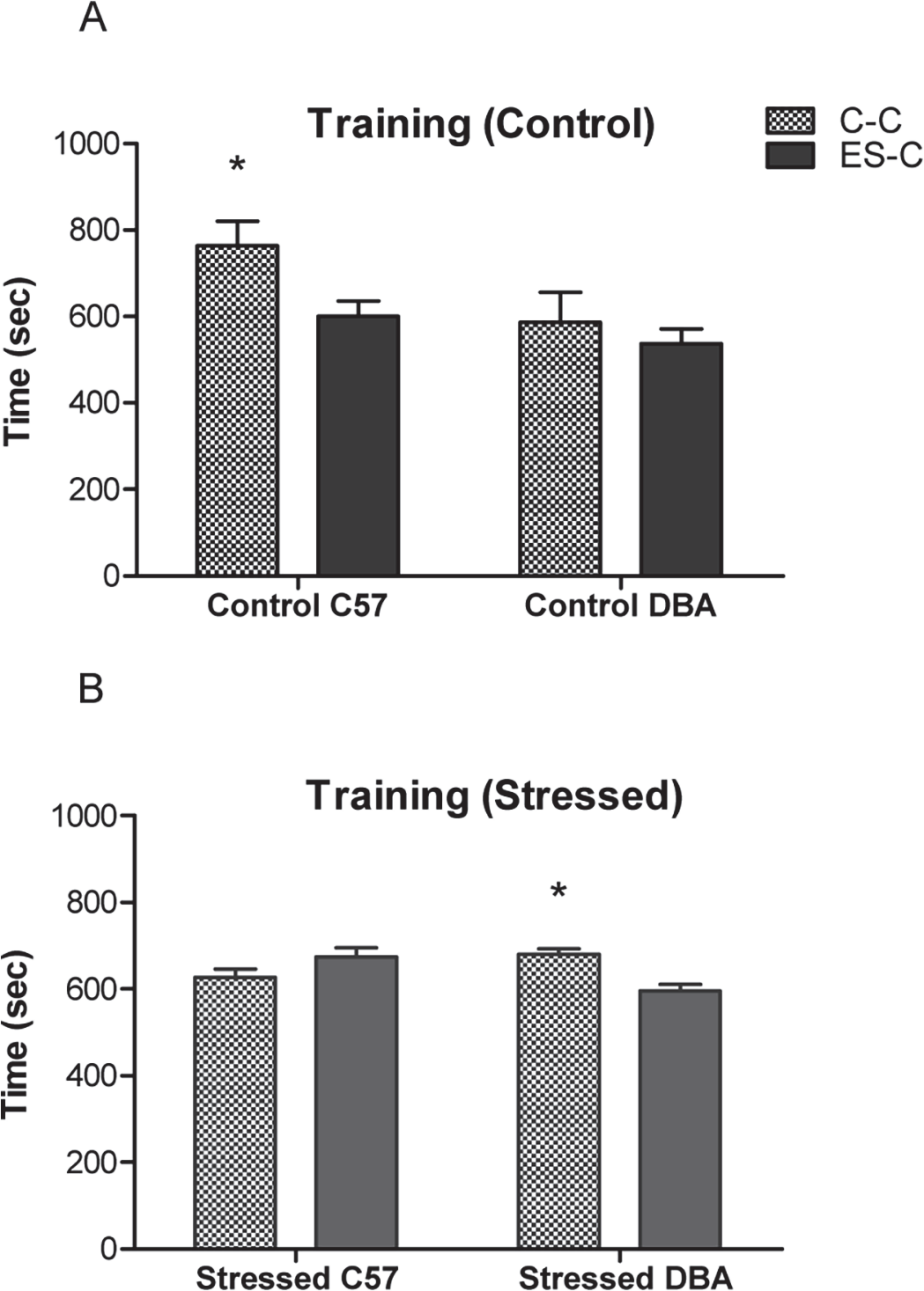
Conditioned Suppression Training in C57 and DBA mice. Time spent (sec ± SE) in the chamber containing chocolate (C-C) and in the empty safe chamber (ES-C) during training phase by Control C57/DBA groups (n = 6 for each group) (A) and Stressed C57/DBA mice (n = 8 for each group) (B). * p< 0.05 in comparison with ES-C.

In the analysis of the training phase, we observed a significant strain x treatment x chamber interaction (F(1,72) = 6.52; p< 0.001). Comparison of the time spent in the C-C and ES-C in each group indicated that only the Control C57 and Stressed DBA groups preferred the C-C versus the ES-C during the training phase (Control C57: F(1,10) = 6.32; p< 0.05; Stressed DBA: F(1,14) = 15.60; p< 0.05) (Fig. 2), spending more time in the C-C than ES-C.

**Fig 3:**
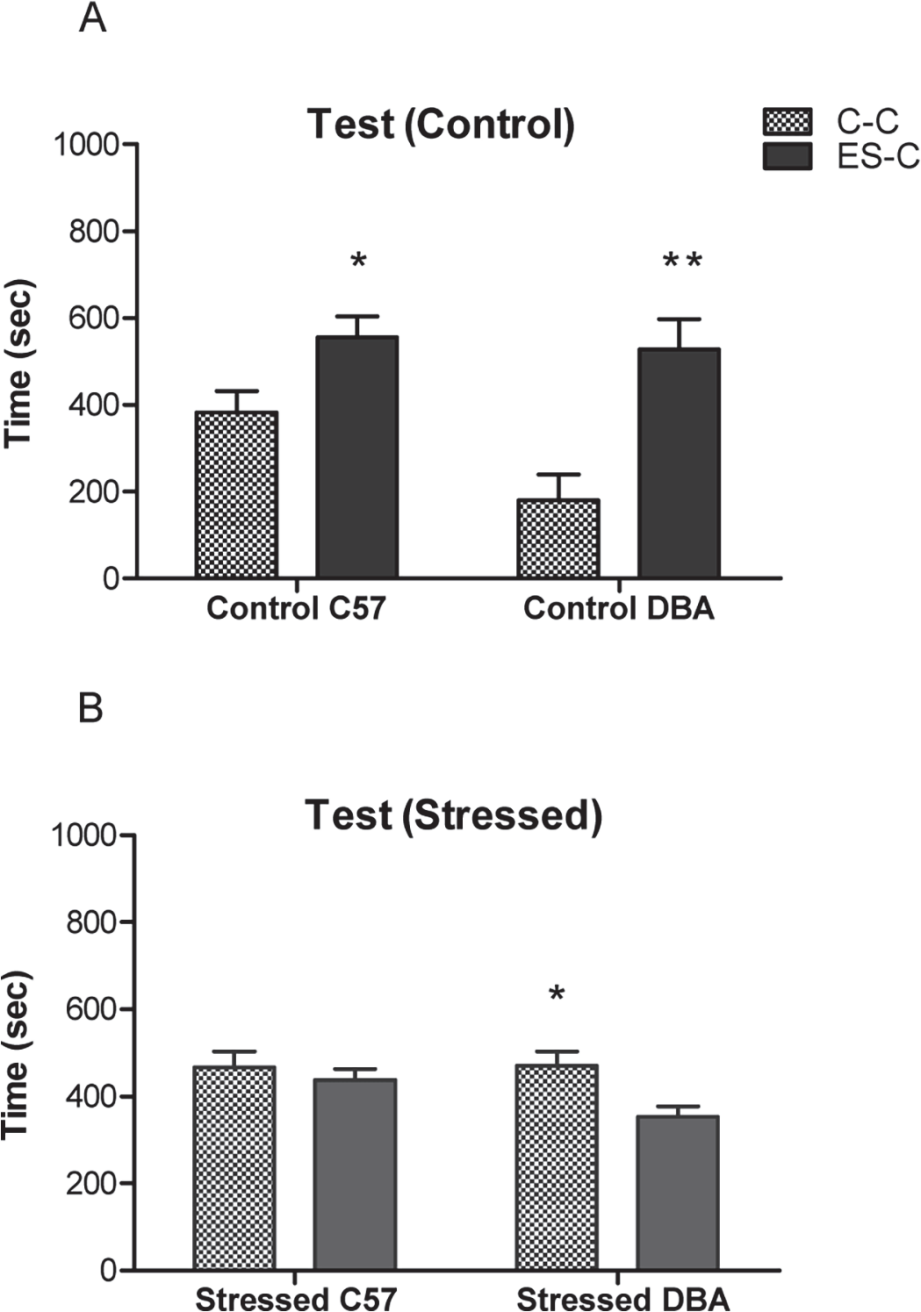
Conditioned Suppression Test in C57 and DBA mice. Time spent (sec ± SE) in the chamber containing chocolate (C-C) and in the empty safe chamber (ES-C) during conditioned suppression test by Control C57/DBA groups (n = 6 for each group) (A) and Stressed C57/DBA mice (n = 8 for each group) (B). * p< 0.05; ** p< 0.01 in comparison with C-C.

Concerning the test results, we observed a significant interaction between strain, treatment and chamber (F(1,72) = 6.0; p< 0.001). The two strains showed different patterns of time spent in the C-C and ES-C. Both control groups (C57, DBA) spent more time in ES-C in comparison with the chamber that contained chocolate (C-C), in which the conditioned stimulus (CS) was present (C57: F (1,10) = 6.04; p < 0.05; DBA: F (1,10) = 12.32; p < 0.01), indicating conditioned suppression of chocolate-seeking during presentation of the CS. In contrast, whereas Stressed C57 mice showed no significant tendency or aversion for either chamber (F (1,14) = .381; n.s.), Stressed DBA animals spent more time in the C-C in comparison with the ES-C, (F (1,14) = 7.38; p< 0.05) (Fig. 3), thus indicating food-seeking behavior despite its possible harmful consequences.

These results indicate that the exposure to our environmental conditions rendered chocolate-seeking impervious to punishment signals, transforming adaptive food-seeking behavior into compulsive seeking only in DBA mice (Fig. 3).

### Chocolate intake and weight

To evaluate the chocolate intake shown by Control and Stressed groups of both strains (Control C57, Control DBA, Stressed C57, Stressed DBA), the consumption of chocolate was assessed during the different phases (pre-exposure, training, test) of the conditioned suppression procedure.

**Fig 4:**
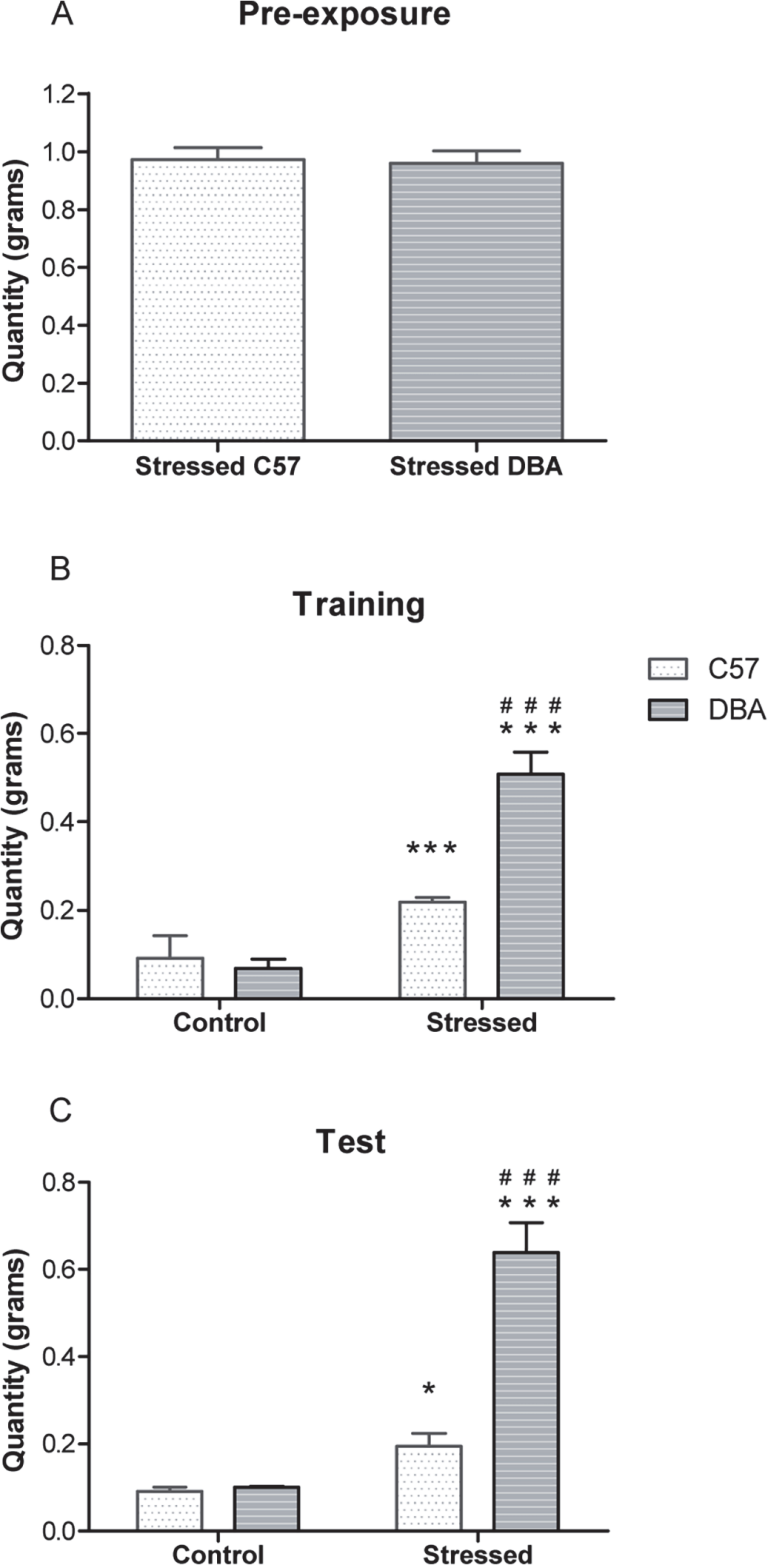
Chocolate intake in C57/DBA Control and Stressed groups. Chocolate intake in C57/DBA Control (n = 6 for each group) and Stressed (n = 8 for each group) animals recorded during pre-exposure (A), training (B), and test (C). Data are expressed as mean grams (overall mean of days ± SE for A and B). * p < 0.05; *** p< 0.001 in comparison with the control group of the same strain. ### p < 0.001 in comparison with the same group of the other strain.

With regard to chocolate intake on pre-exposure phase, there was no significant difference between stressed C57 and Stressed DBA mice (F(1,14) = 0.83; n.s.) (Fig. 4).

With regard to chocolate intake during the training phase, there was a significant interaction between strain and treatment F(1,24) = 20.10; p< 0.001). In the individual between-group comparisons, we noted a significant difference between Control DBA versus Stressed DBA ((F(1,12) = 46.17; p< 0.001), Control C57 versus Stressed C57 ((F(1,12) = 24.25; p< 0.001), and Stressed C57 versus Stressed DBA mice ((F(1,14) = 27.52; p< 0.001) (Fig. 4). Stressed DBA animals showed significantly higher chocolate intake compared to all other groups.

Analysis of chocolate intake on the test day revealed a significant strain x treatment interaction (F(1,24) = 21.48; p< 0.005). Individual between-group comparisons showed a significant difference between control and Stressed DBA ((F(1,12) = 38.49; p< 0.001), Control and Stressed C57 ((F(1,12) = 7.90; p< 0.05) and Stressed C57 and Stressed DBA mice ((F(1,14) = 33.32; p< 0.001) (Fig. 4). Stressed DBA animals experienced significantly greater chocolate intake compared with all other groups, suggesting compulsive chocolate consumption, in agreement with the seeking behavior in the conditioned suppression test.

**Fig 5:**
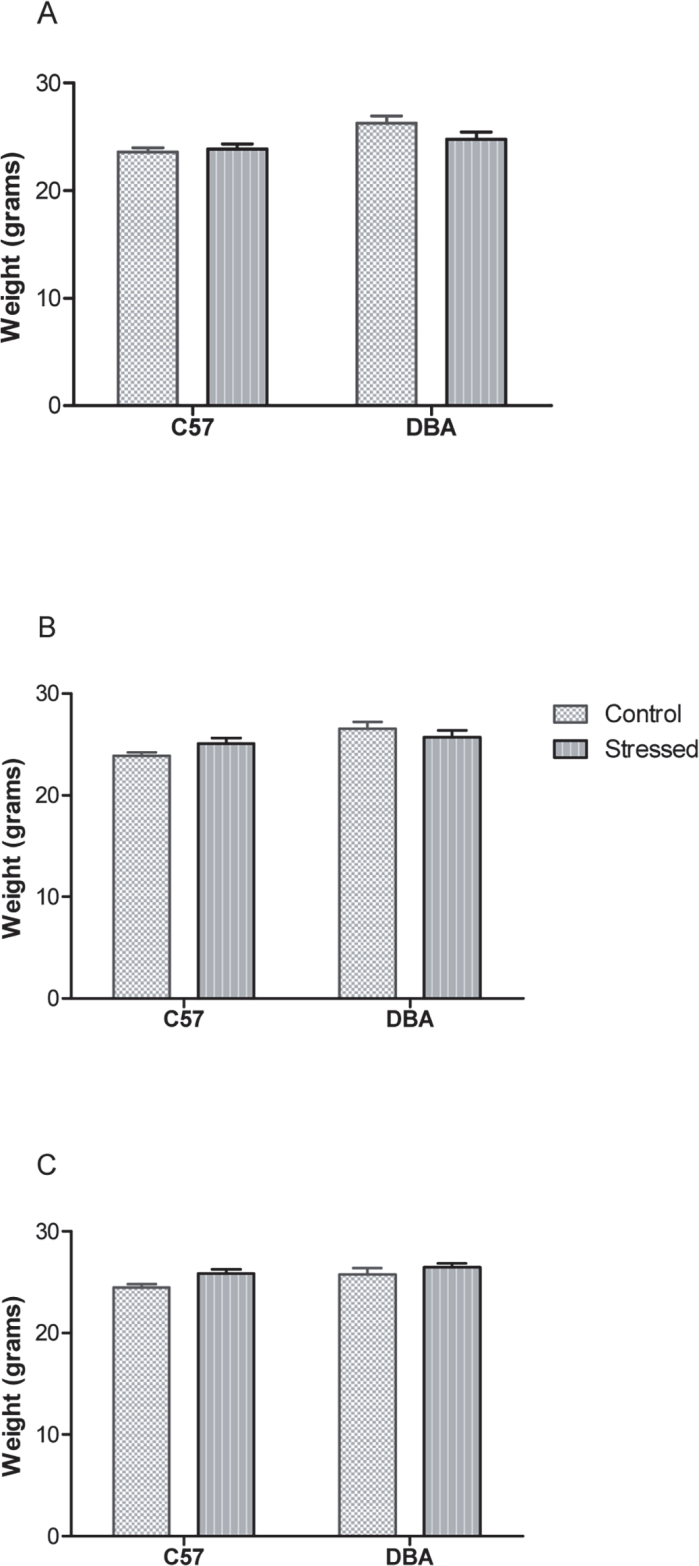
Animal weight. Weight in Control (n = 6 for each group) and Stressed (n = 8 for each group) C57/DBA groups measured before manipulation started (A), on the first training day (B) and on the Test day (C). Data are expressed as gram ± SE.

Finally, concerning weight results, statistical analysis showed that the animals weight did not differ significantly between groups on the first day of the experiment (before the experimental procedure started (F(1,24) = 2.22; ns), on the training phase (F(1,24) = 2.97; n.s.) and on the day of the conditioned suppression test (F(1,24) = 0.58; n.s.) (Fig. 5).

Overall, our data demonstrate a strong interaction between genetic factors and environmental conditions in the expression of compulsive eating, consistent with previous studies that reported a critical function of these factors in certain eating disorders [3–5, 38].

### Dopaminergic and noradrenergic receptor expression in mpFC, NAc, and CP of Stressed DBA vs Control DBA mice

**Fig 6:**
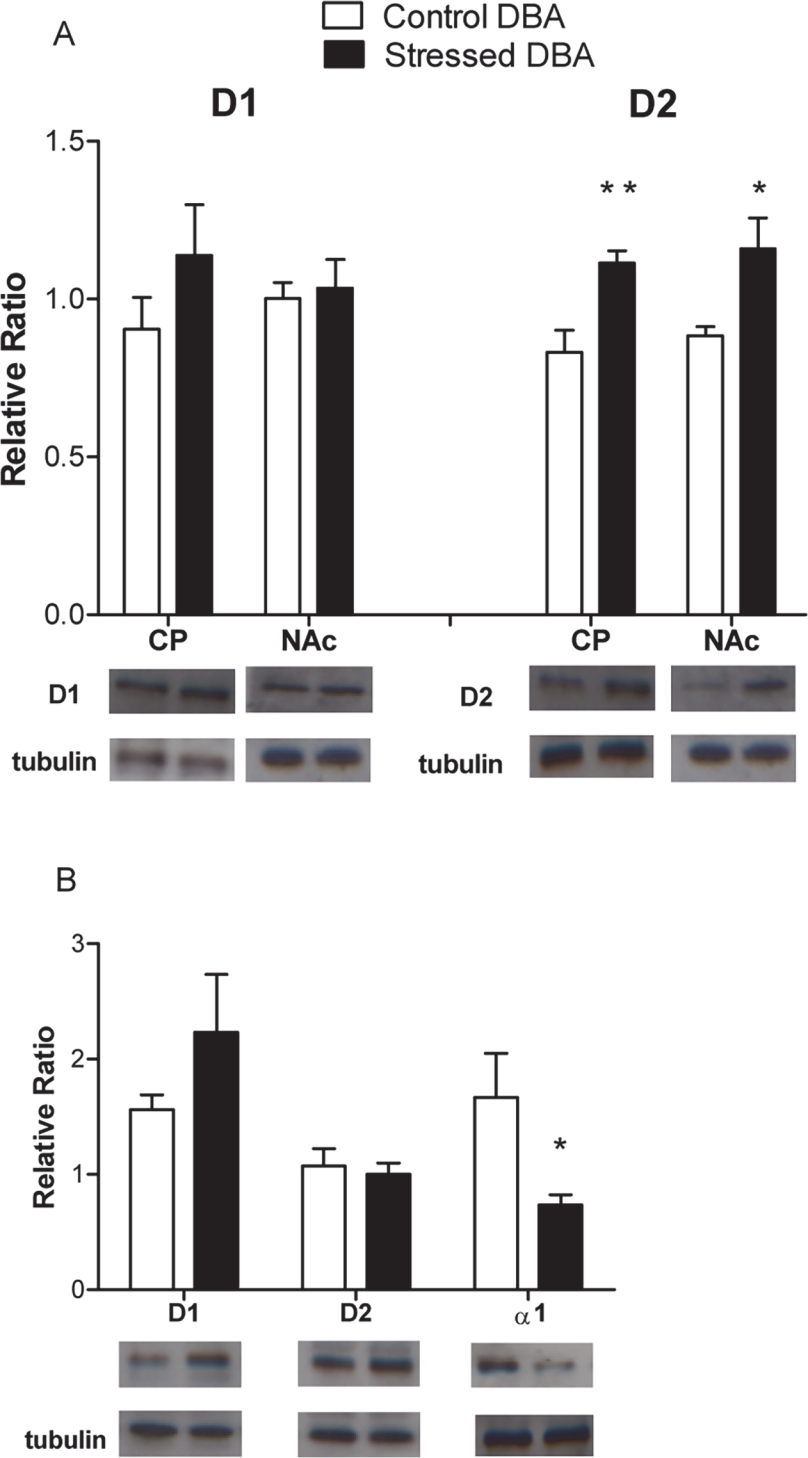
Expression of DA and NE Receptors in DBA strain. Expression of D1R and D2R in CP and NAc (A) and D1R, D2R and α1 in mpFC (B) of Stressed DBA (n = 8) and Control group (n = 6). * p< 0.05; ** p< 0.01 in comparison with control group. Data are shown as relative ratio ± SE.

To assess the expression of dopaminergic and noradrenergic receptors in animal showing compulsion-like eating behavior (Stressed DBA), the expression of α1R, D1R and D2R in the mpFC as well as D1R and D2R in the NAc and CP was evaluated in Stressed vs. Control DBA mice (Fig. 6).

D2Rs were upregulated in the NAc (F(1,12) = 5.58; p< 0.05) and in the CP (F(1,12) = 10.74; p< 0.01) of Stressed DBA compared with Control DBA mice (Fig. 6), indicating a selective effect on striatal D2 receptors in animals showing compulsion-like eating behavior. No significant effect was evident for D1 receptors. α1Rs expression was lower in the mpFC of Stressed DBA group compared to Control DBA mice (F(1,12) = 7.27; p< 0.05) (Fig. 6). No significant effect was observed for prefrontal D1R or D2R receptors expression.

### Dopaminergic and noradrenergic receptor expression in mpFC, NAc, and CP of naïve DBA versus naïve C57 mice

**Fig 7:**
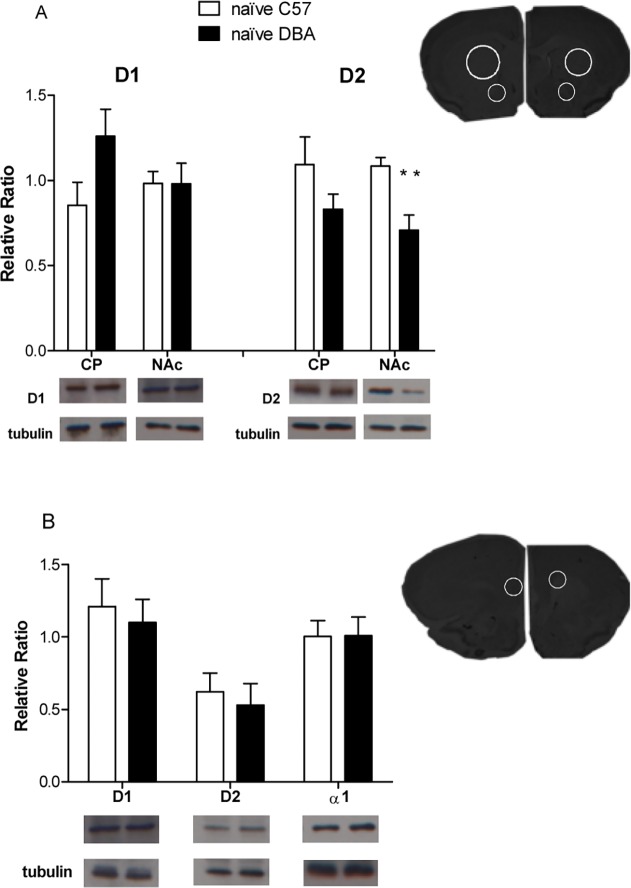
Expression of DA and NE Receptors in naïve C57 and DBA animals. Expression of D1R and D2R in CP and NAc (A) and D1R, D2R, and α1 in mpFC (B) of naïve C57/DBA groups (n = 6 for each group). ** p<0.01 in comparison with naïve group of the other strain. Data are shown as relative ratio ± SE.

In order to evaluate the baseline receptors availability of α1R, D1R and D2R, the expression of α1R, D1R and D2R in the mpFC as well as D1R and D2R in the NAc and CP was evaluated in two different groups of naïve animals of both strains (naïve C57 and naïve DBA) (Fig. 7).

We observed significantly selective lower D2R availability in the NAc of naïve DBA versus naïve C57 mice (F(1,10) = 11.80; p< 0.01). No other significant difference was seen in D1R, D2R, or α1R in the other areas of the brain (Fig. 7). These results, consistent with previous data [4, 54, 60, 61], support the hypothesis that low D2R availability is a “constitutive” risk genetic factor underlying the vulnerability to maladaptive eating.

## Discussion

We assessed compulsive eating in terms of conditioned suppression of palatable food-seeking/intake under adverse conditions [38] in C57 and DBA mice. Exposure to environmental conditions induced compulsion-like eating behavior, depending on genetic background. Moreover, this behavioral pattern appeared to be linked to low availability of accumbal D2 receptors. We also observed D2R upregulation and α1R downregulation in the striatum and mpFC, respectively – a potentially neuroadaptive response that parallel the shift from motivated to compulsion-like eating behavior.

Our experiments suggest that the interaction between access to chocolate pre-exposure and caloric restriction renders chocolate-seeking impervious to signals of punishment, transforming adaptive food-seeking behavior into compulsion-like eating behavior. Notably, this behavior depends strongly on genotype. The conditioned suppression test results indicate that only Stressed DBA animals showed food-seeking behavior, despite possible harmful consequences.

This effect can’t be ascribed to a difference in shock sensitivity between C57 and DBA mice, as shown by the supporting experiment (see S1 Methods and S2 Fig.) and as reported by other groups [62]. Moreover, food-seeking behavior developed, in Stressed DBA animals, in parallel to intake behavior as demonstrated by the high chocolate intake shown by this group. Although consuming large quantities of palatable foods can indicate increased motivation for food, doing so despite harmful consequences, such as tolerating punishment to obtain it, reflects pathological motivation for food (compulsion) [5].

Thus, whereas DBA mice constitute an “ideal model” of resistance to drugs of abuse [24] and food-related disorders under normal conditions (present results), they become most sensitive to drug- [24] and food-related effects when subjected to specific environmental pressures. Moreover, preliminary experiments indicate that exposure to only one of these variables (pre-exposure to chocolate or caloric restriction, separately) fails to induce this phenotype (S1 Methods and S3 Fig.). Thus, only the addictive effect of the environmental conditions (pre-exposure to chocolate and caloric restriction) makes eating behavior refractory to signals of punishment (compulsion-like eating behavior). This result is consistent with evidence that shows that availability of palatable [46, 51], stress exposure [1, 63–65], and a synergistic relationship between stress and calorie restriction are the most important factors that promote eating disorders in humans and animal models [65–67].

The shift from motivated to compulsion-like eating behavior shown by Stressed DBA mice seems to be related to altered dopaminergic and noradrenergic receptors expression in the pFC-NAc-CP circuit. In fact, Stressed DBA mice, which exhibited compulsive eating behavior (as shown by the absence of conditioned suppression), showed an upregulation of D2R in the NAc and CP and a downregulation of α-1AR in the mpFC, compared to control DBA. To rule out that the effects observed could be induced by different amount of chocolate consumption on the test session shown by Control and Stressed DBA, an additional experiment was performed. The experimental conditions and the procedure were as described for Control and Stressed DBA, but receptors expression was performed on the brains removed from mice without chocolate consumption (on the test day). Results from this experiment (S1 Methods and S4 Fig.), clearly exclude that the upregulation of D2R in the NAc and CP as well as the downregulation of α-1AR in the mpFC shown by Stressed DBA can be induced to chocolate consumption.

The results observed in the NAc and CP of Stressed DBA mice do not allow us to determine the effects on DA transmission – i.e., whether the changes increase dopaminergic tone, necessitating more detailed information on the D2 receptor form – e.g., the proportion of the 2 alternative mRNA splice variants, D2R-long (D2L) and D2R-short (D2S) – in the 2 areas, because the relative proportion of the isoforms in the striatum influences neural and behavioral outcomes of D1R and D2/3R co-activation [68–70]. We hypothesize that the increase in postsynaptic receptors and consequent rise in dopamine transmission sustain motivation and invigorate food-seeking behavior [11]. However, more details studies are needed to investigate which type of D2Rs is affected in our experimental procedure.

Increased striatal D2R expression in Stressed DBA mice seems to be in contrast with the hypothesis suggesting that the downregulation of striatal D2R is a neuroadaptive response to the overconsumption of palatable food. However, downregulation of striatal D2R has been reported to be a neuroadaptive response to overconsumption of palatable food and drug intake in humans and animals [4, 44, 60, 71–75] but also a genetic risk factor underlying vulnerability to maladaptive eating [4, 54, 60, 61, 75]. The greater striatal D2R expression that we observed in this study could be the result of a neuroadaptive response to our environmental conditions (pre-exposure, calorie restriction) underlying a specific symptom (compulsive eating) that is shared by other, more complex eating disorders. The debate over this issue has often considered obesity and binge eating disorders, in which complex behavioral patterns (such as increased weight, intermittent feeding episodes, extended access to a high-fat diet) develop—not compulsion-like eating behavior *per se*, as evaluated in this study.

Increasing evidence implicates striatal D1R and D2R in the cost-benefit computation that determines the willingness to expend effort in obtaining a preferred reward, thus affecting motivated behavior [10–14]. Moreover, optimal goal-directed behaviors and motivation appear to correlate with higher D2R levels in the striatum [12, 76–79]. Our study indicates that excessive striatal D2R expression is also linked to a pathological behavioral phenotype, prompting the hypothesis that optimal D2R expression is a neural correlate of ideal goal-directed behaviors and motivation.

Another significant result was the lower availability of D2R in the NAc of naïve DBA versus naïve C57 mice. As discussed, reduced D2R expression has been suggested to be a genetic risk factor of the vulnerability to maladaptive eating [4, 54, 60, 61, 75]. Moreover, decreased D2/D3 dopaminergic receptor availability in the ventral striatum has been proposed to confer an increased propensity to escalate drug intake and correlate with high impulsivity [16, 79, 80]. Further, DBA/2 mice have been reported to have high impulsivity levels [81, 82]. Thus, we speculate that low accumbal D2R availability observed in naïve DBA mice accounts for the disparate inclination toward the development of compulsive eating under specific environmental conditions, such caloric restriction and availability of palatable food—factors that affect the development and expression of eating disorders [4, 46, 64, 83, 84].

We observed decreased prefrontal α1R expression in Stressed versus Control DBA mice. Although prefrontal NE transmission has been suggested to be required for food-related motivated behavior [9] and although NE neurons (in particular through α1Rs) mediate the reinforcing effects of drugs of abuse [57, 58, 85], no study has examined the involvement of prefrontal noradrenergic receptors in compulsion-like eating behavior. Our results extend previous findings on the function of prefrontal NE transmission in food-related motivated behavior, suggesting that specific receptors govern aberrant motivation related to compulsive eating. Downregulation of α1R in the mpFC could be indicative of an adaptive process that underlies the shift from motivated toward compulsive behavior, driven by a faded role of the cortex and a dominant function of the striatum. However, further studies are needed to investigate this hypothesis.

The hypothalamus is one of the most important brain area regulating food-intake [86–88]. However, different brain circuits, other than those regulating hunger and satiety, have been suggested to be involved in food consumption [60, 89]. Moreover, several neurotransmitters and hormones, including DA, NE, acetylcholine, glutamate, cannabinoids, opiods and serotonine, as well as neuroptides involved in homeostatic regulation of food intake, such as orexin, leptin and ghrelin, are implicated in the rewarding effects of food [60, 90–92]. Thus, the regulation of food intake by the hypothalamus seems to be related to different neural circuits processing the rewarding and motivational aspects of food intake [60], such as prefrontal-accumbal system. It's to note that C57 and DBA mice show numerous behavioral differences and the functional and anatomical characteristics of their brain neurotransmitter systems have been extensively examined in these inbred strains [19, 23], thus suggesting a different, strain-dependent, regulation of motivation, reward, learning, and control circuits.

The best-established mechanism involved in processing the rewarding and motivational aspects of food (and drug) is the brain’s dopaminergic reward circuitry [45, 51, 60]. Repeated stimulation of DA reward pathways is believed to trigger neurobiological adaptations in various neural circuits, thus making seeking behavior “compulsive” and leading to a loss of control over one’s intake of food (or drugs) [51, 60].

It has been suggested that under different access conditions, the potent reward-inducing capacity of palatable foods can drive behavioral modification through neurochemical alterations in brain areas linked to motivation, learning, cognition, and decision making that mirror the changes induced by drug abuse [83, 93–99]. In particular, the changes in the reward, motivation, memory, and control circuits following repeated exposure to palatable food is similar to the changes observed following repeated drug exposure [60, 95]. In individuals who are vulnerable to these changes, consuming high quantities of palatable food (or drugs) can disrupt the balance between motivation, reward, learning, and control circuits, thereby increasing the reinforcing value of the palatable food (or drug) and weakening the control circuits [51, 60].

Based on this observation and on results from present study, it can proposed that the shift from motivated behavior to compulsive eating behavior observed in DBA mice could be related to an interplay between genetic vulnerability (low accumbal D2 receptors availability observed in this study as well as differences in other neurotransmitters and hormones involved in food-related brain circuits) and exposure to environmental conditions that, inducing a D2R upregulation and α1R downregulation in the striatum and mpFC, respectively, can lead at an “unbalanced” interaction between circuits that motivate behavior and circuits that control and inhibit pre-potent responses [60, 95].

## Conclusions

There are few studies on gene-environment interaction in human eating disorders [2]. The animal model that we propose here could be used to understand how environmental factors interact with genetic liability and neurobiological factors to promote the expression of compulsion-like eating behavior, also providing new insights into drug addiction.

## Supporting Information

S1 Fig Punching position. Representative position of punching in the medial preFrontal Cortex (mpFC) (A), Nucleus Acumbens (NAc) and Caudate-Putamen (CP) (B).
(TIFF)

S2 Fig Shock sensitivity threshold in C57 and DBA mice. Shock sensitivity in C57 and DBA animals (Methods S1). Mean (μA ± SE) shock threshold observed in C57 and DBA animals.
(TIFF)

S3 Fig Conditioned Suppression Test in DBA mice. Time spent (sec ± SE) in chamber containing chocolate (C-C) empty-safe chamber (ES-C) during Conditioned Suppression Test by DBA pre-exposed and DBA Food Restricted Groups.
(TIFF)

S4 Fig Expression of DA and NE Receptors in DBA mice. Expression of D2 receptors in the CP and NAc as well as of α1 in the mpFC of Stressed and Control DBA mice (n = 6 for each group). * p< 0.05 in comparison with Control group. Data are shown as relative ratio ± SE.
(TIFF)

S1 Methods Supporting Materials and Methods. (DOC)
