# Supplementary figures and images for: When Chocolate Seeking Becomes Compulsion: Gene-Environment Interplay
Source: PLoS One. 2015 Mar 17;10(3):e0120191. doi: 10.1371/journal.pone.0120191 (PMC4363151; doi:10.1371/journal.pone.0120191)

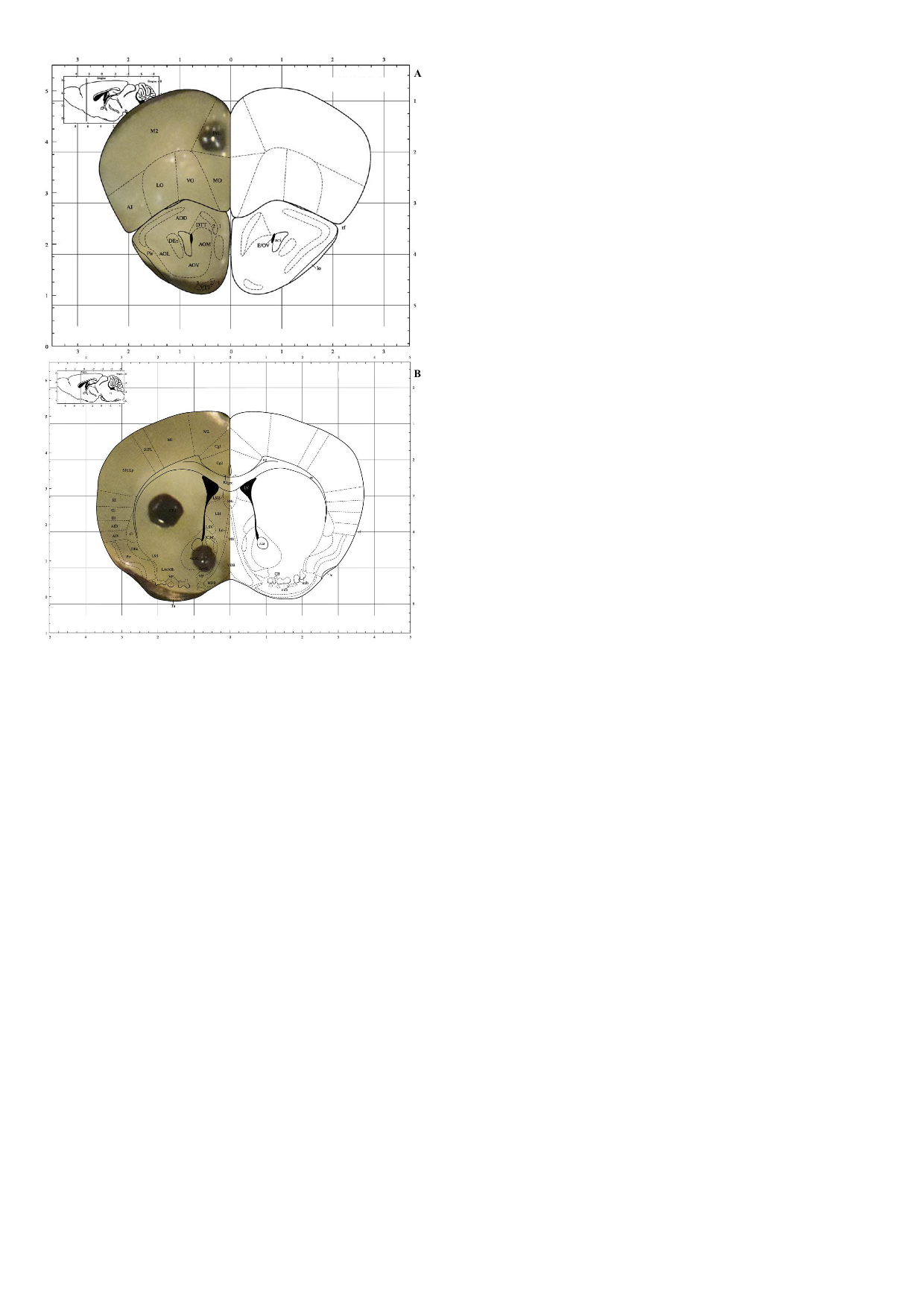

Supplement: S1 Fig — Representative position of punching in the medial preFrontal Cortex (mpFC) (A), Nucleus Acumbens (NAc) and Caudate-Putamen (CP) (B). (TIFF) [file pone.0120191.s001.tiff]

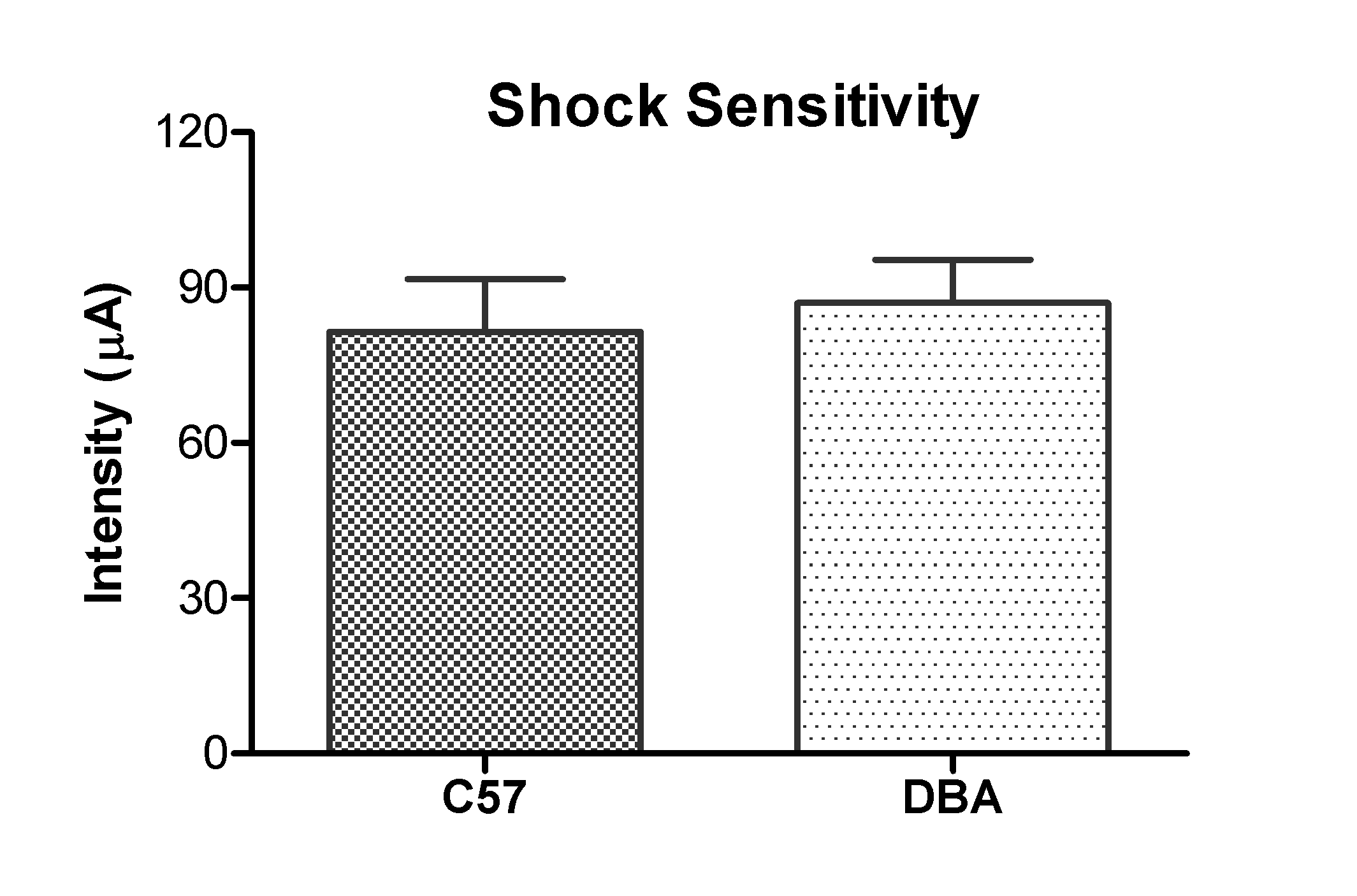

Supplement: S2 Fig — Shock sensitivity in C57 and DBA animals (Methods S1). Mean (μA ± SE) shock threshold observed in C57 and DBA animals. (TIFF) [file pone.0120191.s002.tiff]

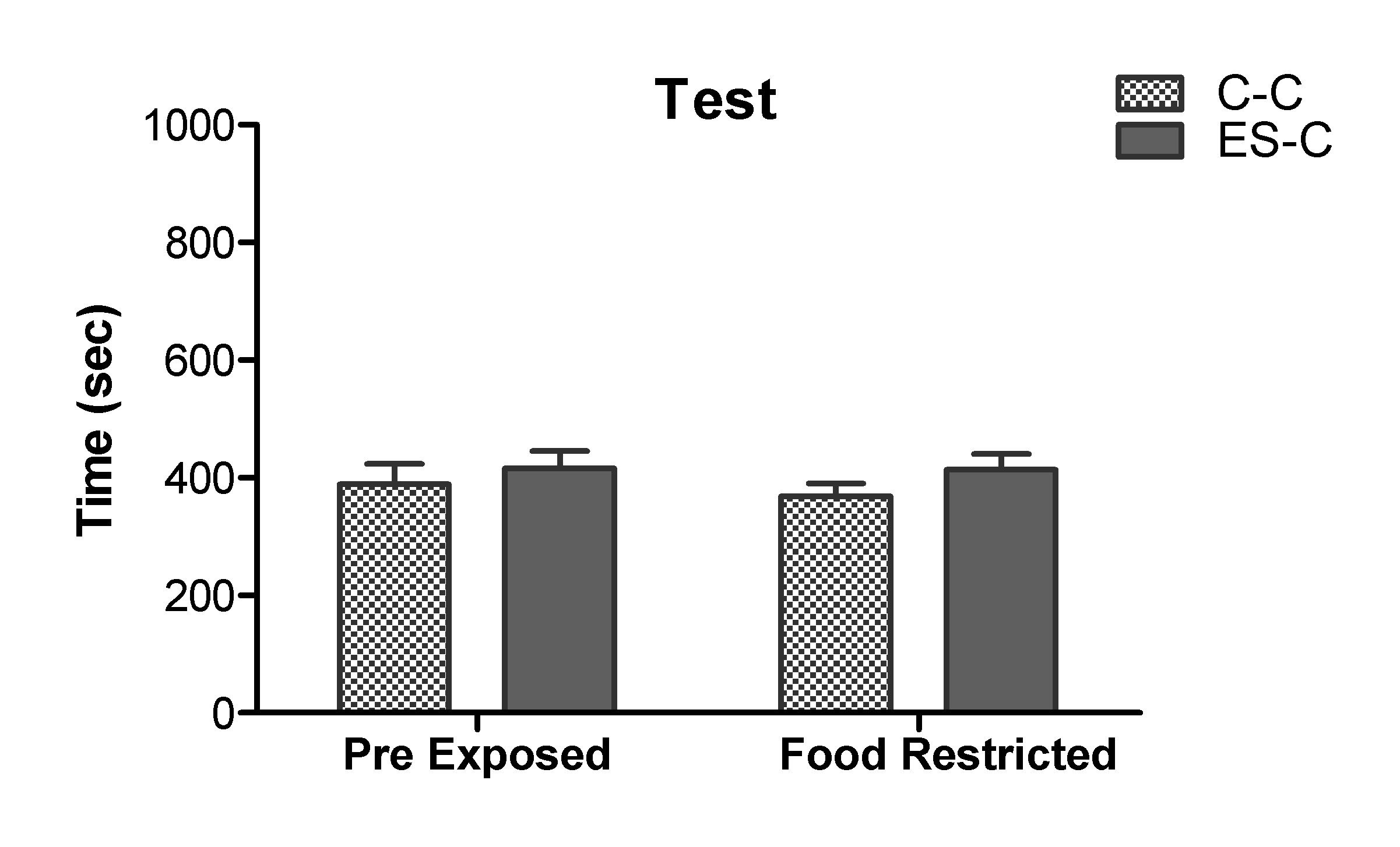

Supplement: S3 Fig — Time spent (sec ± SE) in chamber containing chocolate (C-C) empty-safe chamber (ES-C) during Conditioned Suppression Test by DBA pre-exposed and DBA Food Restricted Groups. (TIFF) [file pone.0120191.s003.tiff]
