# Supplementary material for: When Chocolate Seeking Becomes Compulsion: Gene-Environment Interplay
Source: PLoS One. 2015 Mar 17;10(3):e0120191. doi: 10.1371/journal.pone.0120191 (PMC4363151; doi:10.1371/journal.pone.0120191)
Supplement: S1 Methods — (DOC) [file pone.0120191.s005.doc]

**Supporting Methods**

**Shock Sensitivity**

To rule out differences in sensitivity to foot-shock, C57 and DBA mice (n= 5 for each group) were tested for foot-shock sensitivity. The latter was measured using a modified version of the flinch-jump test (2). Individual mice were placed in the testing apparatus for a 1-2 min acclimation period; no background noise was presented during the testing period. Then, they received 6 series of 6 shocks (1 sec), ranging from 15 to 150 μA, delivered at 20 sec intervals through the grid floor. The series of shocks were delivered in alternating ascending and descending order; the first series was in ascending order. Shock threshold was defined as the lowest shock intensity (μA) at which an animal’ s hind foot left the grid floor. For each mouse, the mean value of shock thresholds recorded in each series was calculated. Statistical analysis revealed no significant difference between C57 and DBA mice (F (1,8 = 0.18 n.s.) (S2 Fig.), in agreement with a previous work (3). Performances of different strains cannot be ascribed to differences in shock sensitivity, because similar mean shock thresholds (μA) were observed in C57 and DBA mice.

**Conditioned Suppression Test in DBA pre-exposed and DBA Food restricted groups**

Two groups of mice were used in this supporting experiment, DBA pre-exposed (n= 8) and DBA food-restricted (FR, n= 8). Repeated-measure ANOVA within each group revealed no significant difference between C-C and ES-C chambers in conditioned Suppression test in both groups (DBA pre-exposed: F (1,14)= 0.34 ns; DBA FR: (F (1,14)= 1.78 ns.) (S3 Fig.).

**Expression of DA and NE Receptors in Stressed and Control DBA mice**

Two groups of mice were used in this supporting experiment, Stressed DBA (n= 6) and Control DBA (n=6). One-way ANOVA revealed a significant difference between the groups: D2-CP (F(1,10)=6.16; p<0.05); D2R-NAc (F(1,10)=5.38; p<0.05), α1-mpFC (F(1,10)=6.85; p<0.05) (S4 Fig.).

**Supporting Reference**

1. Latagliata EC, Patrono E, Puglisi-Allegra S, Ventura R (2010) Food seeking in spite of harmful consequences is under prefrontal cortical noradrenergic control. BMC Neurosci 8: 11-15.

2. Puglisi-Allegra S, Cabib S, Ebel A, Kempf J, Castellano C (1986) Passive avoidance behavior in mice: interaction between age and genotype. Exp Aging Res 12: 107-109.

3. Szklarczyk K, Korostynski M, Golda S, Solecki W, Przewlocki R (2012) Genotype-dependent consequences of traumatic stress in four inbred mouse strains. Genes Brain and Behav 11: 977-985.
